# The Impact of Prolonged Mechanical Ventilation on Overall Survival in Patients With Surgically Treated Brain Metastases

**DOI:** 10.3389/fonc.2021.658949

**Published:** 2021-03-18

**Authors:** Patrick Schuss, Niklas Schäfer, Christian Bode, Valeri Borger, Lars Eichhorn, Frank A. Giordano, Erdem Güresir, Muriel Heimann, Yon-Dschun Ko, Jennifer Landsberg, Felix Lehmann, Anna-Laura Potthoff, Alexander Radbruch, Christina Schaub, Katjana S. Schwab, Johannes Weller, Hartmut Vatter, Ulrich Herrlinger, Matthias Schneider

**Affiliations:** ^1^ Department of Neurosurgery, Center of Integrated Oncology (CIO) Bonn, University Hospital Bonn, Bonn, Germany; ^2^ Division of Clinical Neuro-Oncology, Department of Neurology, Center of Integrated Oncology (CIO) Bonn, University Hospital Bonn, Bonn, Germany; ^3^ Department of Anesthesiology and Intensive Care, University Hospital Bonn, Bonn, Germany; ^4^ Department of Radiation Oncology, Center of Integrated Oncology (CIO) Bonn, University Hospital Bonn, Bonn, Germany; ^5^ Department of Oncology and Hematology, Center of Integrated Oncology (CIO) Bonn, Johanniter Hospital Bonn, Bonn, Germany; ^6^ Department of Dermatology and Allergy, Center of Integrated Oncology (CIO) Bonn, University Hospital Bonn, Bonn, Germany; ^7^ Department of Neuroradiology, Center of Integrated Oncology (CIO) Bonn, University Hospital Bonn, Bonn, Germany; ^8^ Department of Internal Medicine III, Center of Integrated Oncology (CIO) Bonn, University Hospital Bonn, Bonn, Germany

**Keywords:** prolonged mechanical ventilation, brain metastases, overall survival, cancer, intensive care

## Abstract

**Objective:**

Surgical resection represents a common treatment modality in patients with brain metastasis (BM). Postoperative prolonged mechanical ventilation (PMV) might have an enormous impact on the overall survival (OS) of these patients suffering from advanced cancer disease. We therefore have analyzed our institutional database with regard to a potential impact of PMV on OS of patients who had undergone surgery for brain metastases.

**Methods:**

360 patients with surgically treated brain metastases were included. The definition of PMV consisted of postoperative mechanical ventilation lasting for more than 48 hours. Analysis of survival incorporating established prognostic factors such as age, location of BM, and preoperative physical status was performed.

**Results:**

14 of 360 patients with BM (4%) suffered from postoperative PMV after surgical treatment of BM. Patients with PMV presented in a significantly more impaired neurological condition preoperatively than patients without (p<0.0001). Multivariate analysis determined PMV to be a significant prognostic factor for OS after surgical treatment in patients with BM, independent of other predictive factors (p<0.0001).

**Conclusions:**

The present study demonstrates postoperative PMV as significantly related to poor OS in patients with surgically treated BM. Postoperative PMV is a so far underestimated prognostic predictor, but might be utilized for optimized patient management early in the postoperative phase. For this purpose, the results of the present study should encourage the initiation of further scientific efforts.

## Introduction

Compared to previous decades, modern intensive care medicine provides a significant survival advantage for critically ill patients due to a continuous gain in knowledge and further refined technology (1). Patients with a diagnosis of cancer and the need for mechanical ventilation (e.g., due to respiratory failure) have a grim prognosis, as numerous previous studies have documented (2–4).

Patients with brain metastases (BM) are referred to the group of patients with advanced-stage cancer (5). As such, they often have already undergone multiple debilitating surgeries and other treatments. In the case of BM with brain surgery necessary to reduce intracranial mass, these vulnerable patients are again exposed to a wide range of possible complications (6). In the case of a resulting prolonged need for mechanical ventilation, in addition to the physical challenges, the necessary adjuvant therapy may be postponed with devastating consequences - as recently demonstrated in patients with glioblastoma and meningioma (7, 8). It is therefore imperative to address the impact of prolonged mechanical ventilation (PMV) on cancer patients and especially those with BM. Academic debate on potential prognostic factors (such as PMV) might contribute to a better allocation of patients as well as consultation of family members and might avoid vain medical care and subsequent inferior end-of-life quality (9). Despite various studies indicating the impact of PMV in different cancer patients, we are unaware of any preceding detailed research evaluating a possible prognostic effect of PMV in patients suffering from BM.

Consequently, the aim of the present study was to determine the impact of postoperative PMV on overall survival (OS) in patients with BM.

## Materials and Methods

### Patients

All patients with surgically treated BM between 2013 and 2018 were entered into a computerized database (SPSS, version 25, IBM Corp., Armonk, NY). The institutional ethics committee has granted approval for this study.

After identification of eligible patients, different information such as patient characteristics, radiological features, and functional neurological status were collected at admission and analyzed in the following. Here, the Karnofsky Performance Score (KPS) was used for preoperative evaluation of patients according to their neurological functional status. For further analysis, the results were dichotomized and thus a KPS ≥ 70 was defined as favorable outcome. Patients with BM were further stratified into two groups according to the classification of the American Society of Anesthesiologists (ASA), respectively patients with preoperative ASA 1 or 2 and patients with preoperative ASA ≥ 3. The overall preoperative burden of comorbidity in the patients studied was assessed using the Charlson Comorbidity Index (CCI), with additional points added for age to adjust for age (10). Age-adjusted CCI ≥ 10 was utilized as the dichotomization threshold, as previously established (6).

Individual treatment decisions were made at the initial presentation of the patient and during follow-up by the weekly institutional interdisciplinary tumor advisory board meetings for the Central Nervous System, as described previously (6).

Due to the lack of published data in surgically treated brain metastasis patients, PMV was deemed to be a postoperative invasive ventilation lasting more than 48 hours, as previously reported and defined in another patient population (11, 12).

Overall survival (OS) was defined as the period from the day of BM surgery until death or last observation. Patients in whom no further follow-up information was available due to further off-site treatment were excluded from further analysis. All parameters were compared in relation to OS.

### Statistics

To perform data analysis, the computer software package SPSS (version 25, IBM Corp., Armonk, NY) was used. Unpaired categorical and binary variables were analyzed in contingency tables using the Fisher’s exact test. OS was analyzed by the Kaplan-Meier method. Relevant clinical factors were entered into a multivariable Cox proportional risk model to predict overall survival. Results with p<0.05 were considered statistically significant.

## Results

### Patient Characteristics

At the neuro-oncological center of the authors, a total of 388 patients were surgically treated for BM from 2013 to 2018. 28 patients (7%) were excluded from further due to the lack of follow-up information. Therefore, 360 patients with surgically treated BM were included in further analysis. The median age was 65 years (range 22-91 years). Patients with surgically treated BM presented with a median KPS score of 80 at admission. Overall, 14 patients with surgically treated BM suffered from postoperative PMV (4%). The median duration of postoperative mechanical ventilation for all patients with surgically treated BM was 8.8 hours (Interquartile range [IQR] 7-19). Median OS for patients with surgically treated BM was 9 months (95% CI 7.1-10.9).

### Patients With BM and PMV

In total, 14 patients (4%) with surgically treated BM developed PMV during the course of postoperative treatment. Patients who experienced PMV after surgery presented significantly more often with a KPS <70 on admission than patients without PMV (8/14 [57%] versus [vs.] 39/346 [11%]; p<0.0001, OR 10.5). Moreover, patients who developed postoperative PMV after surgery for BM were preoperatively significantly more often evaluated as ASA ≥ 3 compared to patients with surgically treated BM without PMV (13/14 [93%] vs. 190/346 [55%]; p=0.005, OR 10.6). Furthermore, patients with postoperative PMV presented with a preoperative increased comorbidity burden (age-adjusted CCI ≥ 10) compared to patients without postoperative PMV (14/14 [100%] vs. 258/346 [75%]; p=0.03, OR 1.1). Evidence of extracranial disease did not differ significantly between patients with and without PMV (8/14 [57%] vs. 157/346 [45%]; p=0.4). Patients who suffered from postoperative PMV exhibited a significantly longer median length of hospital stay (LOS; 24 days; IQR 14-36) compared with patients without PMV (10 days; IQR 7-16; p=0.003). Further details on potential influencing factors in patients with PMV are given in **Table 1**.

**Table 1:** Patient characteristics.

	Patients without PMV (n=346)	Patients with PMV (n=14)	p-value
Median age at surgery (yrs)	64.5	66	p=0.52
Female sex	167 (48%)	9 (64%)	p=0.28
Median preoperative KPS	80	60	p<0.0001
ASA score ≥ 3	190 (55%)	13 (93%)	p=0.0047
Preoperative CCI ≥ 10	258 (75%)	14 (100%)	p=0.03
Primary tumor location			
Lung	147 (42%)	7 (50%)	p=0.59
Breast	44 (13%)	1 (7%)	p=1.0
Melanoma	37 (11%)	2 (14%)	p=0.66
Other	118 (34%)	4 (29%)	p=1.0
Multiple BM	109 (32%)	6 (43%)	p=0.39
Infratentorial location of BM	103 (30%)	7 (50%)	p=0.14
30 days mortality	12 (3%)	8 (57%)	p<0.0001
12 months mortality	194 (56%)	13 (93%)	p=0.005
Median postoperative ventilation time (hrs)	8.4 (IQR 7-12)	136.1 (IQR 78-416)	p<0.0001
Median OS (mo)	10	0	p<0.0001

ASA, American society of anesthesiologists; BM, brain metastases; CCI, Charlson Comorbidity Index; hrs, hours; IQR, interquartile range; mo, months; OS, overall survival; PMV, prolonged mechanical ventilation; yrs, years.

### Influence of PMV on Overall Survival and Mortality

**Figure 1:**
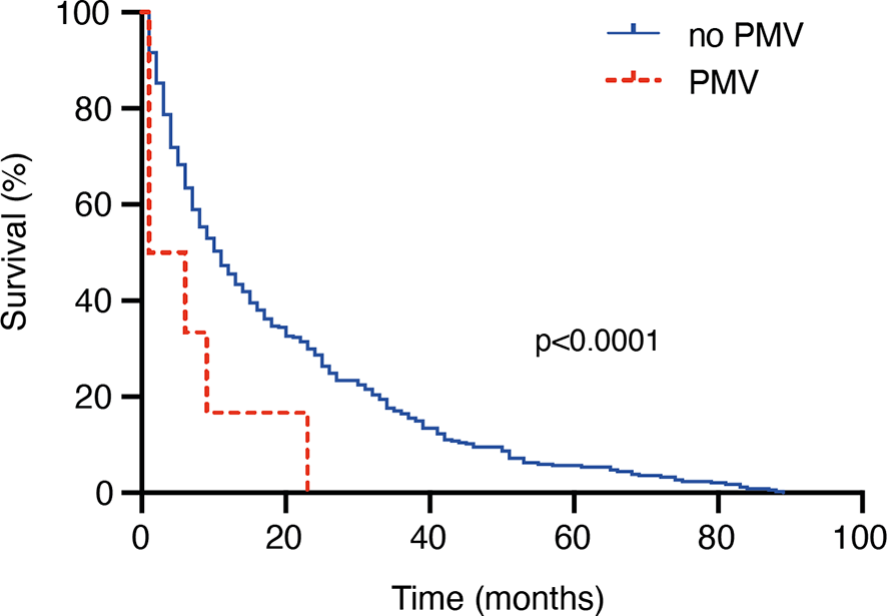
PMV is associated with poor overall survival in patients with surgically treated BM.

Patients with surgically treated BM who suffered from postoperative PMV achieved a median OS of < 1 months, whereas patients without PMV achieved an OS of 10 months (95% CI 8-12; p<0.0001; **Figure 1**). In-hospital mortality was 50% in patients with PMV compared to 1% in patients without (7/14 vs. 3/346; p<0.0001, OR 114). 71% of patients with postoperative PMV suffered from additional postoperative complication within 30 days after surgery (10/14). Specifically, two patients with PMV experienced postoperative bleeding (14%), one patient with PMV required surgery for cerebrospinal fluid leakage (7%), while 4 patients with PMV suffered cardiac and/or pulmonary complications (28%). Discharge destinations for patients with postoperative PMV after surgical resection of BM were transfer to another acute-care hospital in 2 patients (14%), home in 2 patients (14%), to a rehabilitation institution in also 2 patients (14%), and to a nursing home/palliative care in one patient (7%). Mortality at 30 days was significantly higher in patients with surgically treated BM suffering from postoperative PMV compared to those patients without PMV (8/14 [57%] vs. 12/346 [3%]; p<0.0001, OR 37.1). Furthermore, mortality after 1 year significantly differed between patients without and with PMV (194/346 [56%] vs. 13/14 [93%]; p=0.0052, OR 10.2).

### Multivariate Analysis

**Figure 2:**
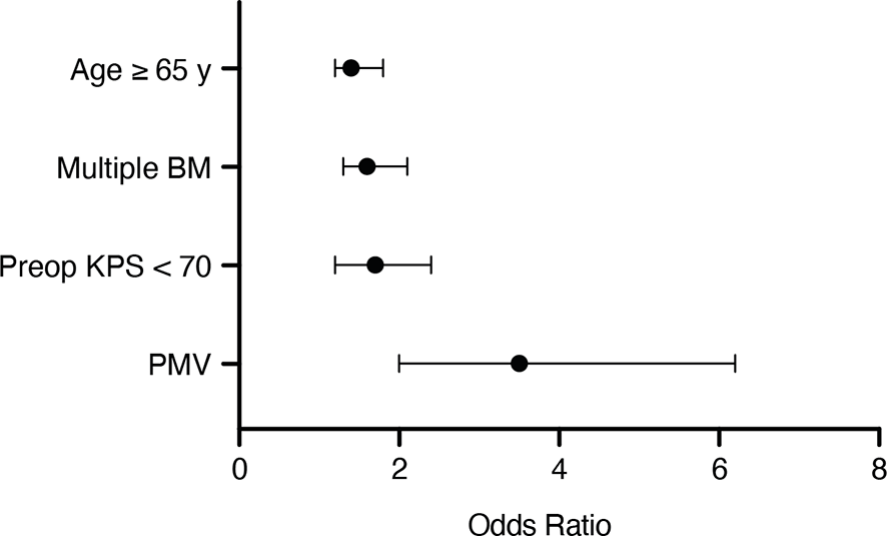
Results from the multivariate analysis.

We conducted a multivariate survival analysis using a proportional hazards regression analysis (Cox regression model) to determine independent predictors of OS in patients with surgically treated BM. The multivariate analysis revealed the variables “age ≥ 65 years” (p=0.002, OR 1.4, 95% CI 1.2-1.8; **Figure 2**), “multiple BM” (p<0.0001, OR 1.6, 95% CI 1.3-2.1), “preoperative KPS < 70” (p=0.002, OR 1.7, 95% CI 1.2-2.4) and “PMV ≥ 48 hours” (p<0.0001, OR 3.5, 95% CI 2.0-6.2) to be significant and independent predictors for a poor OS after surgical treatment of BM.

## Discussion

The present study demonstrates PMV to constitute an independently significant prognostic factor for dismal OS in patients with BM after surgical treatment.

Precedently, in patients suffering from different malignancies, PMV had been described as an impactful prognostic parameter. A large database-based study previously reported the 1-year survival rate in cancer patients in need of PMV to be as low as 14% (4). However, in the case of patients suffering from BM, these patients are referred to as patients with advanced-stage cancer (5, 13). Furthermore, previous reports on the impact of PMV on cancer patients are not specifically focused on this subgroup of severely ill, advanced-stage cancer patients. Nevertheless, patients with BM are not necessarily treated surgically: Especially in the case of small, non-space-occupying and/or multiple findings, other treatment options are primarily established (14, 15). However, with an appropriately indicated surgical therapy, whether for histological confirmation and/or cytoreduction, these vulnerable patients must endure another pronounced surgical trauma (6). However, patients with BM pose an additional challenge in the management of cancer patients presenting with critical illness. In the case of patients with surgical treated BM, a postoperative necessary PMV must not solely be related to reduced physical resources and/or underlying diseases (e.g. lung cancer), but can also be triggered by the consequences of the localization of the surgical treated BM and/or its associated postoperative complications (7, 8). Nevertheless, significantly more patients with an increased preoperative comorbidity burden (assessed using CCI) suffered postoperative PMV in the present patient cohort. However, in addition to common factors contributing to a postoperative complex respiratory weaning situation, patients with intracranial tumors (here: BM) bear the additional likelihood of disease-induced impaired vigilance, which may in itself result in the need for ventilation. The unsatisfactory survival of patients with BM and postoperative PMV in the present study might also be attributed to a delay of postoperative adjuvant treatment and/or further therapy of the underlying cancer disease caused by active intensive care treatment.

Although emerging directly postoperatively and thus initially still in concordance with the patient’s wishes, PMV might under certain circumstances lead to an (undesired) prolonged stay in the intensive care unit (ICU). In the present study, a preoperatively impaired KPS and an increased ASA score were shown to be possible predictors for the subsequent need for postoperative PMV in patients with surgically treated BM. Although these are associated factors that are unlikely to be improved preoperatively, early identification of patients at risk may help in assessing the optimal extent of postoperative monitoring as well as in the preoperative dialogue with the affected patients and/or their family members regarding the realistic expectations of the neurosurgical procedure. As is increasingly the case with regard to postoperative monitoring of many elective craniotomies, the admission of cancer patients to an ICU is a very controversial topic of discussion, partly due to the lack of appropriate guidelines (16, 17). During a prolonged ICU stay of cancer patients, communication with family members and/or (depending on vigilance) with the patient herself/himself, plays an important role. In order to act in the best interest of the patient, it is advisable for (neuro)oncologists and intensive care physicians to jointly discuss and guide the ICU treatment of cancer patients (16). The (neuro)oncological assessment of the prognosis and the possible further therapy options/prospects can thus be supplemented by the intensive care medical expertise of what is feasible in intensive care. It is important for all treating physicians to make sure that the extensive intensive care options are in the (presumed) interest of the patient concerned.

With regard to the above-mentioned highly sensitive decisions to be made, the authors are certain that the present study, in identifying postoperatively required PMV as an independent predictor of poor survival in patients with BM, could offer a decisive contribution or at least raise awareness of this (rare but devastating) condition in patients with surgically treated BM.

### Limitations

Along with the inherently retrospective aspect of data collection, the present study has further limitations. For example, the patients were not randomized but treated according to the individual consensus of the treating physicians. In addition, the relevant patient group to be described is very small, so that the underlying causes of PMV can be inferred only with difficulties and reluctance. Furthermore, the small number of patients does not allow a detailed correlation of ventilation time and the underlying months of survival. Moreover, the heterogeneous group design with respect to the primary tumor, the associated different onward treatment strategies and again the incomplete record of all intriguing study aspects (postoperative epilepsy, adjuvant therapies) in all patients makes further evaluation prone to error, respectively. Nevertheless, the present study investigates this aspect for the first time in surgically treated BM patients and thus provides the basis for initiating further studies and correlation analyses.

## Conclusions

Postoperative PMV is a yet underestimated independent predictor for poor OS following neurosurgical treatment in patients with BM. Therefore, greater emphasis should be dedicated to patients with surgically treated BM requiring postoperative PMV, prompting the need for continued research on the early detection of PMV.

## Data Availability Statement

The original contributions presented in the study are included in the article/supplementary material. Further inquiries can be directed to the corresponding author.

## Ethics Statement

The studies involving human participants were reviewed and approved by Local ethics committee at the University of Bonn. Written informed consent for participation was not required for this study in accordance with the national legislation and the institutional requirements.

## Author Contributions

Conceptualization: PS and MS. Methodology: PS, NS, MH, UH, and MS. Data collection: PS, NS, MH, A-LP, JW, and MS. Statistics: PS, MS, and A-LP. Figures: MS, A-LP, and PS. Writing—original draft: PS and MS. Study supervision: PS, UH, and MS. Proofreading: all authors. All authors contributed to the article and approved the submitted version.

## Conflict of Interest

The authors declare that the research was conducted in the absence of any commercial or financial relationships that could be construed as a potential conflict of interest.
